# Single vesicle imaging indicates distinct modes of rapid membrane retrieval during nerve growth

**DOI:** 10.1186/1741-7007-10-4

**Published:** 2012-01-30

**Authors:** Jacob H Hines, Steven J Henle, Lucas P Carlstrom, Mohammad Abu-Rub, John R Henley

**Affiliations:** 1 Department of Neurologic Surgery, Mayo Clinic, Rochester, MN, USA; 2 Department of Pediatrics, University of Colorado Anschutz Medical Campus, Aurora, CO, USA; 3 Network of Excellence for Functional Biomaterials, National University of Ireland, Galway, IRL; 4 Department of Physiology and Biomedical Engineering, Mayo Clinic, Rochester, MN, USA

## Abstract

**Background:**

During nerve growth, cytoplasmic vesicles add new membrane preferentially to the growth cone located at the distal tip of extending axons. Growth cone membrane is also retrieved locally, and asymmetric retrieval facilitates membrane remodeling during growth cone repulsion by a chemorepellent gradient. Moreover, growth inhibitory factors can stimulate bulk membrane retrieval and induce growth cone collapse. Despite these functional insights, the processes mediating local membrane remodeling during axon extension remain poorly defined.

**Results:**

To investigate the spatial and temporal dynamics of membrane retrieval in actively extending growth cones, we have used a transient labeling and optical recording method that can resolve single vesicle events. Live-cell confocal imaging revealed rapid membrane retrieval by distinct endocytic modes based on spatial distribution in *Xenopus *spinal neuron growth cones. These modes include endocytic "hot-spots" triggered at the base of filopodia, at the lateral margins of lamellipodia, and along dorsal ridges of the growth cone. Additionally, waves of endocytosis were induced when individual filopodia detached from the substrate and fused with the growth cone dorsal surface or with other filopodia. Vesicle formation at sites of membrane remodeling by self-contact required F-actin polymerization. Moreover, bulk membrane retrieval by macroendocytosis correlated positively with the substrate-dependent rate of axon extension and required the function of Rho-family GTPases.

**Conclusions:**

This study provides insight into the dynamic membrane remodeling processes essential for nerve growth by identifying several distinct modes of rapid membrane retrieval in the growth cone during axon extension. We found that endocytic membrane retrieval is intensified at specific subdomains and may drive the dynamic membrane ruffling and re-absorption of filopodia and lamellipodia in actively extending growth cones. The findings offer a platform for determining the molecular mechanisms of distinct endocytic processes that may remodel the surface distribution of receptors, ion channels and other membrane-associated proteins locally to drive growth cone extension and chemotactic guidance.

## Background

During the construction of neural circuits, growing axons of developing neurons extend long distances en route to the appropriate synaptic targets. New membrane and materials are added to extending axons and increase the plasma membrane surface area by 10 to 1,000-fold [1]. A typical 1-μm-diameter axon extends at the rate of 0.5 mm per day, necessitating the insertion of new membrane at the rate of 1.1 μm^2 ^per minute. The body of evidence indicates that new membrane is incorporated primarily at the motile tip of the axon, the nerve growth cone [2-5]. Cytoplasmic vesicles, derived in the neuronal cell body, undergo anterograde transport along the length of the axon and are added by local exocytosis within the growth cone [6-9].

In addition to a role in axon extension, numerous studies have demonstrated that regulated membrane trafficking is intricately involved in growth cone chemotaxis. Axon pathfinding toward the synaptic target requires dynamic interactions with the extracellular matrix (ECM) and the detection of spatial guidance cues within the local environment. The ability of growth cones to adapt to a wide range of guidance cue concentrations may involve regulated vesicle trafficking [10]. Moreover, attractive growth cone turning toward a locally applied gradient of nerve growth factor requires asymmetric membrane insertion at the attractant side, or leading edge [11]. In contrast, repulsive growth cone turning requires endocytic pathways and correlates with asymmetric endocytosis [12-14]. These findings have led to the notion that the balance of exocytic and endocytic activities across the growth cone serves to control local membrane protrusion versus membrane removal and drives bidirectional axon guidance [15]. Further support for this idea comes from the finding that growth cone collapse is associated with regulated membrane retrieval [16,17].

During axon extension, the growth cone surface membrane is also retrieved at rates sufficient to turn over completely within 30 minutes [18]. At first glance, these energetically demanding processes appear to counteract the substantial membrane addition that must occur in order to drive axon extension. Numerous reports indicate that local membrane retrieval and recycling play important roles in axonal growth [19-24]. The precise regulators of these endocytic routes are incompletely understood, but likely include actin, cholesterol, Pincher, phosphoinositide 3-kinase (PI3K) and Rac1 [19]. Despite these functional insights, the mechanisms by which local membrane retrieval and recycling facilitate nerve growth and guidance are relatively unknown [25]. In chemotaxing cells, mounting evidence indicates that regulated vesicle trafficking, both endocytic and exocytic, is critical for directed migration [26-29]. The emerging view from multiple studies suggests that polarized endocytosis and exocytic recycling can spatially polarize receptor signaling, cytoskeletal regulators, and focal adhesion turnover in order to drive cell motility [30,31].

How endocytic processes regulate membrane dynamics and remodel the growth cone surface membrane to support nerve growth remain outstanding issues that await further characterization of the membrane retrieval processes in the growth cone. Previous ultrastructural studies have provided high-resolution snapshots, revealing coated and noncoated vesicles, membrane-contiguous vacuoles, elongated tubules and stacks of lumenless membrane disks with largely unknown functions [32-36]. However, the identification and discrimination of parallel structures in live growth cones by fluorescence and DIC microscopy has been unyielding [18,35,37-40]. This may reflect the temporal limitations of pulse-chase endocytic assays, which effectively track the fate of endosomes minutes after internalization but fail to capture early events. Here, we have overcome these limitations by local and transient application of lipophilic membrane dyes to single growth cones, combined with high-speed confocal microscopy, in order to monitor the initial formation of single nascent endocytic vesicles. This approach provides high spatiotemporal resolution and has revealed individual modes of rapid endocytic membrane retrieval with distinct spatial distribution and temporal dynamics in actively extending *Xenopus *spinal neuron growth cones. We discovered hot-spots of concentrated single vesicle membrane retrieval events in the growth cone that are actin-dependent, and internalization by much larger endocytic tubules. Furthermore, we have uncovered evidence for unexpectedly rapid recycling of endocytic compartments, where nascent vesicles and tubules disappear within seconds of forming. Finally, we provide evidence that substrate-stimulated outgrowth enhances the rate of bulk endocytosis in the growth cone and requires the function of Rho GTPases. This set of findings and the optical imaging approach may serve as an important foundation for future studies aimed at elucidating the molecular regulators, specific cargo and functional significance of distinct membrane retrieval and recycling pathways in axonal growth and guidance.

## Results

### Detection of single-vesicle retrieval events

**Figure 1:**
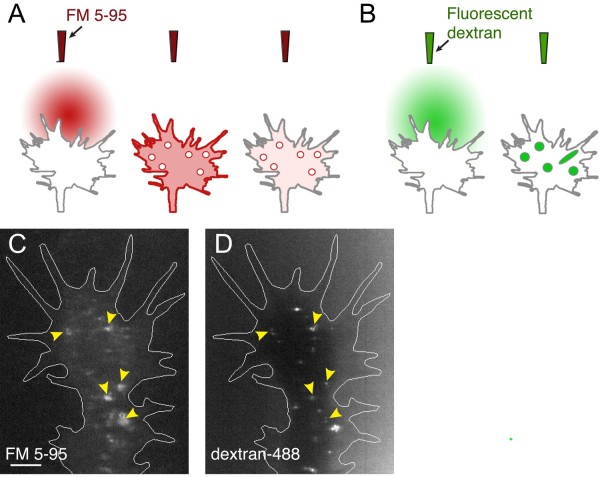
**Focal endocytic assay**. **(A) **Illustration of the focal FM 5-95 membrane labeling assay. A focal pulse delivered from a micropipette containing FM 5-95 labels the growth cone plasma membrane. During subsequent membrane retrieval events, the lipophilic dye is trapped inside nascent vesicles. The surface membrane destains rapidly, revealing single endocytic vesicles. **(B) **Using a similar approach as in (A), fluorescent dextran is focally applied from a micropipette. Seconds later, a second micropipette containing buffered saline washes away the uninternalized fluorescent dextran, revealing nascent dextran-containing endocytic vesicles. **(C-D) **Confocal images of the same growth cone show nascent dye-containing vesicles 10 s after the focal pulse. The labeling micropipette contained both FM 5-95 (C) and Alexa488 conjugated dextran (D). The yellow arrowheads indicate endocytic vesicles exhibiting strong co-labeling. Scale bar, 5 μm.

To visualize membrane retrieval by endocytic vesicles in actively extending growth cones, we transiently labeled the plasma membrane of individual *Xenopus *spinal neuron growth cones with the lipophilic membrane dye FM 5-95 while performing time-lapse confocal microscopy. A micropipette positioned directly in front of the growth cone trajectory delivered a focal dye pulse to label the surface membrane. Local dye diffusion and the fast destaining properties of FM 5-95 quickly decreased the fluorescence intensity of surface-bound dye to levels below that of nascent dye-labeled vesicles. As a result, rapid confocal imaging allowed the detection of single endocytic vesicle retrieval events that formed within 5 to 20 s of the initial dye pulse (Figure 1A). Imaging fluorescent beads of various known sizes confirmed the ability to optically resolve individual spheres to at least 96 nm in diameter with the apparent point resolution limited to 400 nm (data not shown).

A similar focal labeling approach applied fluorescent dextran, which is taken up with fluid into the lumen of endocytic vesicles during membrane retrieval (Figure 1B). After the dextran labeling, a second micropipette delivered buffered saline to wash away uninternalized dextran. This attenuated the background fluorescence significantly while leaving the internalized signal unaffected. The consequent increased signal to noise ratio permitted the detection of nascent endocytic vesicles and tubules by time-lapse confocal imaging. When co-applied from the same micropipette, both FM 5-95 and fluorescent dextran revealed numerous nascent vesicles in the growth cone following the brief application period (Figure 1C, D). These findings highlight the rapid kinetics of membrane retrieval in the growth cone by single endocytic vesicles.

### Hot-spots of rapid membrane retrieval in the growth cone

**Figure 2:**
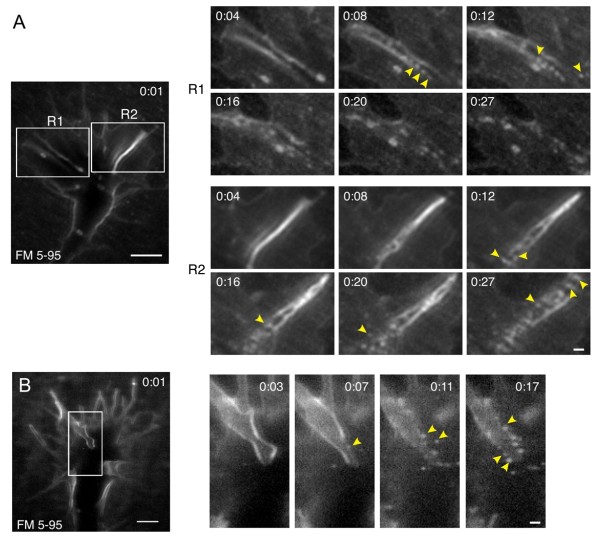
**Small endocytic vesicle hot-spots at dorsal ridges of the growth cone**. **(A-B) **Time-lapse confocal images of FM 5-95 internalization show numerous small endocytic vesicles that form at dye-labeled dorsal ridges of the growth cone. For each example (A-B), the frame showing initial membrane labeling immediately following the focal dye pulse is shown at the left (0:01). The boxed regions (left panels) are shown at higher magnification in the right panels. Time, following the initial membrane labeling, is indicated for each frame in minutes:seconds. In each example, the peripheral filopodia are slightly below the dorsal focal plane in the confocal section. Note the radial progression (inside-out) of endocytic vesicle formation, which is indicated by the yellow arrowheads marking nascent vesicles. Time-lapse movies are shown in Additional file 1. Scale bars, 5 μm (left), 1 μm (right).

**Figure 3:**
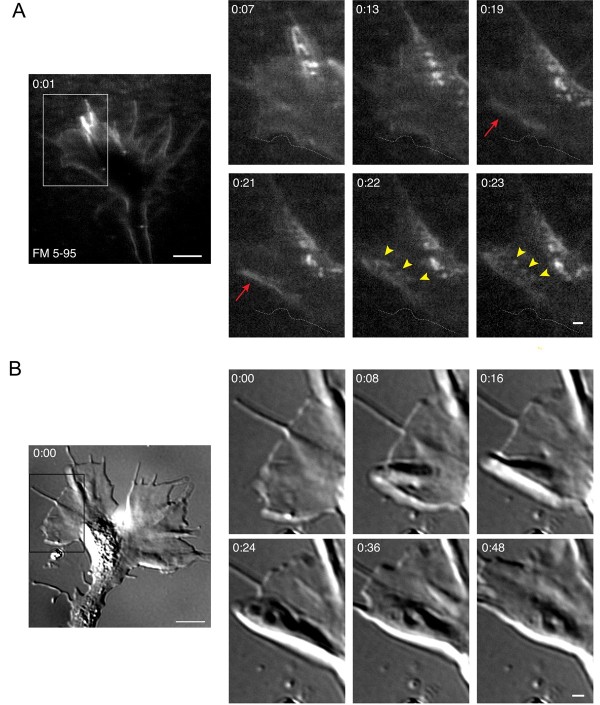
**Small endocytic vesicle hot-spots at the lateral margins of lamellipodia**. **(A) **Time-lapse confocal images of FM 5-95 internalization show endocytosis at the lateral margin of a lamellipodium as the growth cone narrows. The boxed area is magnified in the panels on the right and the time after the initial membrane labeling (minutes:seconds) is indicated in each frame. The static dashed line represents the lamellipodial margin at t = 0:07. In subsequent frames, the lamellipodium remodels to form a dye-rich membrane ruffle (red arrows, 0:19, 0:21). Within seconds, numerous small vesicles form (yellow arrowheads, frames 0:22, 0:23). The corresponding time-lapse movie is shown in Additional file 2. **(B) **Similar ridge formation at a lamellipodial lateral margin observed by time-lapse DIC microscopy. See Additional file 3 for the corresponding time-lapse movie. Scale bars, 5 μm (left), 1 μm (right).

**Figure 4:**
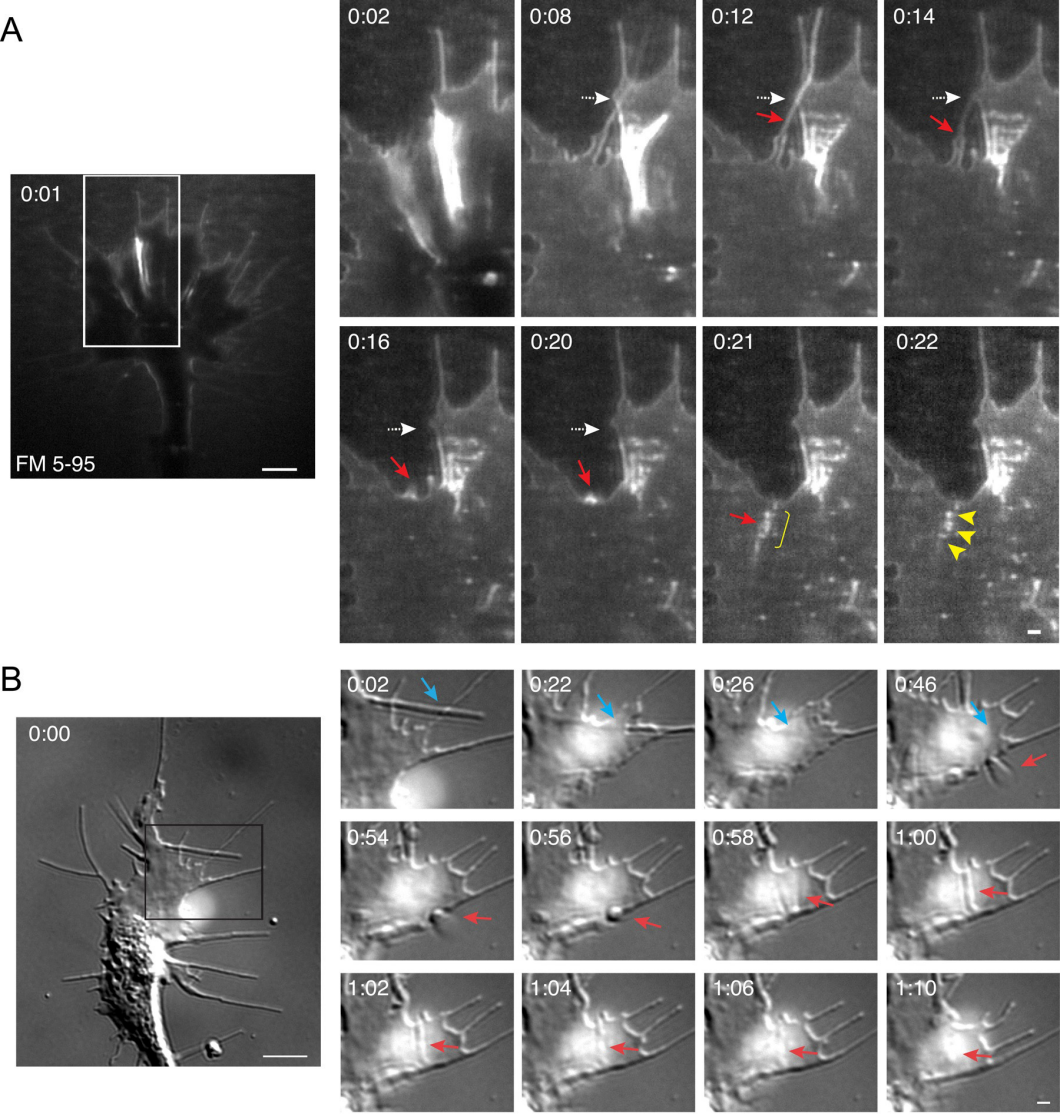
**Small vesicle endocytic hot-spots triggered by filopodial - lamellipodial contacts**. **(A) **Time-lapse confocal images of FM 5-95 internalization show a detached filopodium collapse atop the dorsal surface of the growth cone and trigger an endocytic hot-spot. The boxed region is magnified in the right panels and the time after the initial membrane labeling (minutes:seconds) is indicated in each frame. The dashed arrows (white) are static and indicate the initial position of the filopodium. The detached filopodium lifts out of the focal plane (0:15 to 0:20, red arrows) and within seconds collapses atop the growth cone body (yellow bracket, 0:21), eliciting a streak of small vesicles (yellow arrowheads, 0:22). See Additional file 4 for the corresponding time-lapse movie. **(B) **Time-lapse DIC images show a similar loss of filopodia atop the dorsal surface of the growth cone. Detached filopodia (blue and red arrows) approach lamellipodia and rapidly disappear. One filopodium (red arrow) lifts out of the focal plane (0:46 to 56), collapses atop a lamellipodium (0:58 to 1:06), and disappears as in (A). The corresponding time-lapse movie is shown in Additional file 5. Scale bars, 5 μm (left), 1 μm (right).

**Figure 5:**
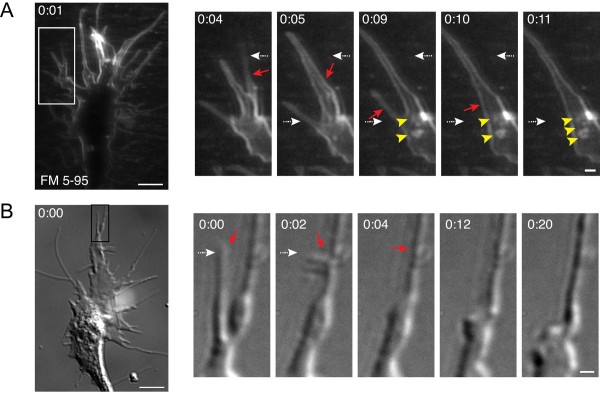
**Small vesicle endocytic hot-spots triggered by filopodial contact**. **(A) **Time-lapse confocal images of FM 5-95 internalization show contact and apparent fusion between adjacent filopodia (red arrows). The boxed area is magnified in the panels at the right and the time after the initial membrane labeling (minutes:seconds) is indicated in each frame. The dashed arrows (white) are static and indicate the initial position of filopodia. Filopodial contact and fusion is accompanied by the formation of multiple small endocytic vesicles at the proximal region of the resulting filopodium (yellow arrowheads). See Additional file 6 for the corresponding time-lapse movie. **(B) **Time-lapse DIC images show similar filopodial-filopodial contacts (red arrows) as in (A). The dashed arrows (white) depict the initial filopodial position. The corresponding time-lapse movie is shown in Additional file 7. Scale bars, 5 μm (left), 1 μm (right).

**Figure 6:**
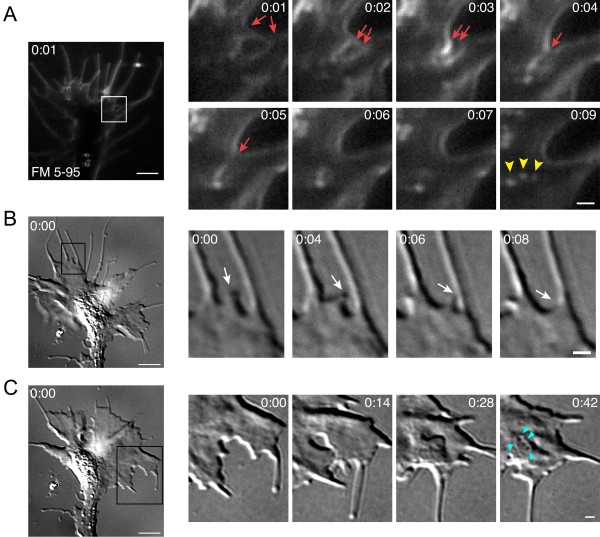
**Small vesicle endocytic hot-spots triggered by lamellipodial contact**. **(A) **Time-lapse confocal images of FM 5-95 internalization show an endocytic hot-spot where adjacent processes contact one another. The boxed area is magnified in the panels at the right and the time after the initial membrane labeling (mintes:seconds) is indicated in each frame. Lamellipodial contacts near the base of filopodia (red arrows) result in the formation of several small vesicles (yellow arrowheads) and subsequent membrane reshaping. For the corresponding time-lapse movie see Additional file 8. **(B-C) **Time-lapse DIC images show similar lamellipodial contacts and membrane remodeling as in (A). Note the contact between adjacent membrane processes (B, white arrows). The blue arrowheads indicate structures highly reminiscent of the reverse shadowcast vacuoles reported by Dailey and Bridgman [35]. See Additional file 9 for the time-lapse movie corresponding to Figure 6B, C. Scale bars, 5 μm (left), 1 μm (right).

**Figure 7:**
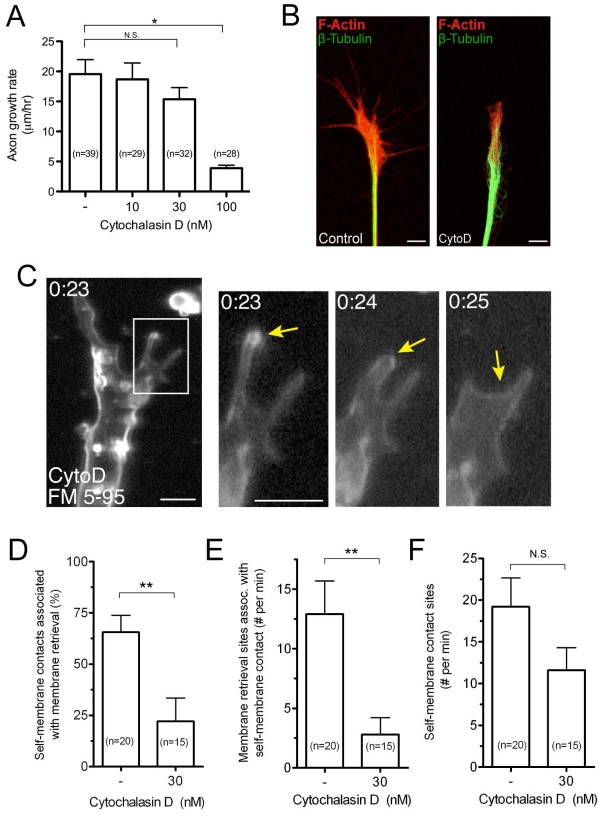
**Endocytic vesicle formation at self-membrane contacts requires F-actin**. **(A) **Summary of axon growth rates in vehicle treated controls and after treatment with cytochalasin D during a one-hour growth assay. Data are the mean ± standard error of the mean and the number of axons measured is indicated for each condition. N.S. (no significant difference), *P *> 0.05, *P < 0.0001, One-way ANOVA, Tukey's post-test. (**B**) Representative confocal images of control (DMSO) and cytochalasin D (CytoD, 30 nM) treated growth cones show the distribution of F-actin and microtubules, as detected by Alexa555-phalloidin (red) and anti-β-tubulin immunolabeling (green). Note the fewer peripheral processes and reduced F-actin after CytoD. Scale bar, 5 μm. (**C) **Time-lapse confocal images after a FM 5-95 dye pulse show a motile filopodium (yellow arrows) contacting and fusing with the growth cone peripheral plasma membrane. The self-membrane contact fails to trigger vesicle formation. The boxed region is magnified in the right panels and the time (minutes:seconds) after the initial membrane labeling is indicated in each frame. The corresponding time-lapse movie is shown in Additional file 10. Scale bars, 5 μm. (**D-E**) Quantitative analysis of membrane retrieval at self-membrane contact sites (filopodial - lamellipodial and filopodial - filopodial contacts) in untreated and cytochalasin D-treated (30 nM) growth cones. The percentage of total self-membrane contact sites associated with membrane retrieval is shown in (D), and the frequency of membrane retrieval events (per minute) occurring at self-membrane contact sites is shown in (E). (**F**) Frequency (per minute) measurements for the total number of self-membrane contact events, including those not associated with membrane retrieval, measured during the focal membrane labeling assays. For (D-F), data are the mean ± standard error of the mean and the number of growth cones is indicated for each bar. N.S., *P *> 0.05, **P < 0.01, *t*-test.

**Figure 8:**
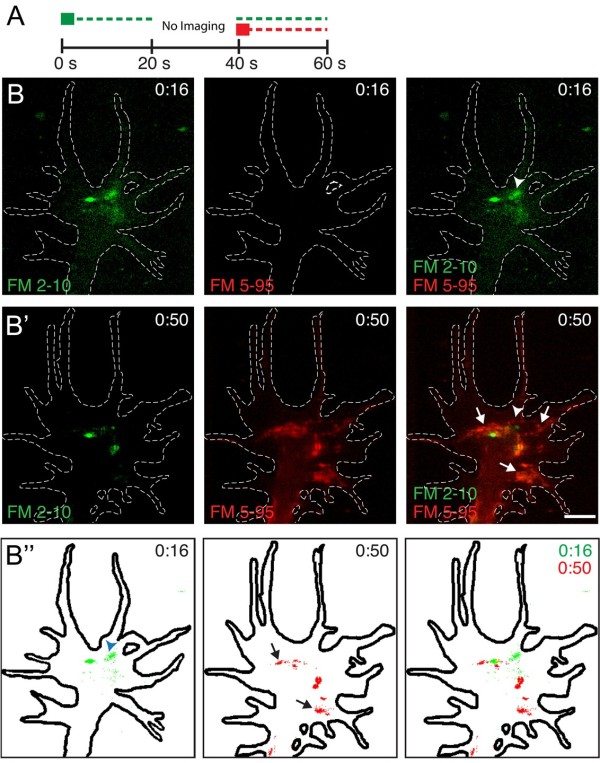
**Sequential dye labeling demonstrates temporally distinct endocytic zones**. (**A) **Illustration of the dual FM dye membrane labeling assay. A micropipette first applied a focal pulse of FM 2-10 (green square, t = 0 s) and confocal images were collected for 20 s. After an additional 20 s interval, a second micropipette applied a focal pulse of FM 5-95 at time 40 s (red square) and confocal images were collected for an additional 20 s. The broken lines depict the presence of the respective FM dyes. (**B-C) **Time-lapse confocal images of a representative growth cone subjected to the dual FM dye labeling assay described in (A). (**B) **Confocal images of FM 2-10 internalization following the initial dye pulse, applied at t = 0:00, show labeled endocytic structures in the growth cone. The white arrowhead marks an endocytic zone. Time (minutes:seconds) following the FM 2-10 pulse is depicted in each frame. (**B'**) Confocal images of the same growth cone following a second dye pulse (FM 5-95, red) show dye labeled nascent vesicles, most of which were not labeled by the prior FM 2-10 pulse. Importantly, the majority of nascent vesicles labeled by FM 5-95 (right panel, white arrows) formed in locations spatially distinct from regions where FM 2-10-positive vesicles had formed (right panel, white arrowhead). Time (min:s) following the initial FM 2-10 dye pulse is indicated in each frame. (**B''**) Binary images, generated from the fluorescence images in B (0:16) and B' (0:50), show distinct endocytic zones during the two FM dye pulses applied at 40-s intervals. See Additional file 11 for the time-lapse movie corresponding to B-B''. Scale bars, 5 μm.

To determine the spatial and temporal dynamics of membrane retrieval during growth cone migration, we used high-resolution confocal microscopy and collected images at 1 Hz in real time during the focal endocytic assay after FM dye labeling. Remarkably, we discovered that single vesicle membrane retrieval events often clustered at hot-spots, defined as focal regions of the growth cone where multiple vesicles formed in near synchrony. The frequency of vesicle formation concentrated within endocytic hot-spots greatly exceeded that seen in surrounding regions of the growth cone surface membrane. By carefully observing endocytic events in multiple growth cones, we were able to identify discrete endocytic modes based on the spatial distribution and temporal dynamics (Figures 2, 3, 4, 5, 6, 7, 8). The nascent endocytic vesicles associated with hot-spots were small (< 0.5 μm diameter). Temporally, the initiation of hot-spots was stochastic. Furthermore, single endocytic vesicles formed at hot-spots during only a short time period lasting up to several seconds following initiation, after which endocytic activity abruptly terminated. In many instances, the induction of endocytic hot-spots correlated with regions of self-contact or occurred at regions undergoing rapid membrane remodeling.

### Membrane retrieval at dorsal ridges and lamellipodial margins

We frequently observed hot-spots of small endocytic vesicles at dorsal ridges of the growth cone (Figure 2). In all cases, endocytic vesicle formation at dorsal ridges originated at the central-most region of the ridge and proceeded radially into the growth cone periphery (Figure 2A, B, Additional file 1). These endocytic "waves" resulted in a streak of nascent vesicles analogous to beads on a string. In total, 65% of the growth cones tested demonstrated endocytosis at dorsal ridges during the brief 20 to 25 s endocytic assay. Notably, dye labeling of the surface membrane was sometimes non-uniform and dorsal ridges showed specific dye enrichment. This may reflect membrane processes extending into the z-plane, or alternatively that FM 5-95 preferentially incorporates into specific membrane domains. We observed endocytosis at most, but not all (87%, 27/31) of the labeled dorsal ridges, suggesting that endocytic activity is most likely not induced by the FM dye in this assay. The high frequency of endocytic activity within dorsal ridges observed during the brief endocytic assay suggests that dorsal ridges are specialized regions of the growth cone that undergo rapid membrane turnover.

Confocal microscopy also revealed hot-spots of labeled small vesicles at the lateral margins of lamellipodia (Figure 3). Lamellipodial sheets, which were initially uniformly labeled with FM 5-95, generated brightly labeled membrane ridges at the outer-most margins of growth cones. Waves of small endocytic vesicles immediately followed this membrane remodeling while membrane ridges disappeared (Figure 3A, Additional file 2). In comparison to dorsal ridges, membrane retrieval at lamellipodial margins was relatively infrequent and was observed in 15% of the growth cones tested. This may reflect the peripheral composition of individual growth cones, which have varying amounts of lamellipodia and filopodia at any given time-point. Among the growth cones tested, only 65% had appreciable lamellipodia at lateral margins where endocytosis could have occurred. Time-lapse DIC microscopy revealed remarkably similar membrane dynamics at the lateral margins of lamellipodia (Figure 3B, Additional file 3). The prevalence of these events varied between growth cones and we routinely observed undulating waves of ridge formation and collapse at lateral margins. These waves were transient and recurred every one to two minutes (data not shown). Because the FM dye labeling assay was carried out for < 30 s, it is inherently improbable for transient waves of this frequency to be captured during this short optical imaging window. This may explain why membrane retrieval at lamallipodial margins was detected in only 15% of the growth cones tested.

### Membrane retrieval at sites of cell-cell contact

The initiation of small vesicle hot-spots as detected by FM dye labeling was commonly associated with self-contact between peripheral extensions of the growth cone. Figure 4 shows endocytic hot-spots that were triggered when a single filopodium contacted the growth cone dorsal surface. The filopodium that had lost substrate attachment momentarily left the focal plane (Figure 4A). Continuing this trajectory, the filopodium then rapidly collapsed atop the growth cone body, triggering the formation of numerous small vesicles along the length of this cell-cell contact (Figure 4A, Additional file 4). We observed membrane retrieval that was initiated at sites of filopodial - lamellipodial contact in 55% of the growth cones tested. Correspondingly, time-lapse DIC microscopy revealed a similar disappearance of detached filopodia after making contact with the growth cone body (Figure 4B, Additional file 5).

Contact between adjacent filopodia also triggered small vesicle endocytic hot-spots, as revealed by FM dye labeling (Figure 5). In some instances, these self-interactions resulted in the apparent fusion of multiple filopodia into a single filopodium. Meanwhile, we observed numerous small vesicles that formed near the proximal base of the new filopodium (Figure 5A, Additional file 6). In the 20 growth cones tested, we observed 35 filopodial fusions, 23 of which (66%) were associated with simultaneous vesicle formation. Filopodial-filopodial fusions were also evident by time-lapse DIC imaging (Figure 5B, Additional file 7), although by this method we cannot rule out the possibility that what appears to be one new filopodium may actually be two tightly bundled filopodia.

The FM dye labeling assay revealed frequent hot-spots of small endocytic vesicles at cell-cell contact sites between adjacent lamellipodial processes (Figure 6). These interactions commonly occurred near the base of filopodia where adjacent sheets of plasma membrane fused together. Numerous nascent vesicles formed concomitantly with membrane remodeling (Figure 6A, Additional file 8). Endocytic hot-spots at lamellipodial contacts occurred in 60% of the growth cones tested. Furthermore, we commonly observed lamellipodial contact-induced endocytic hot-spots at multiple locations of the same growth cone during the focal endocytic assay (data not shown). Similar membrane rearrangements at lamellipodial-lamellipodial contacts were also evident by time-lapse DIC imaging (Figure 6B, C, Additional file 9).

The mechanisms driving the membrane rearrangements and vesicle formation at sites of self-membrane contact are unknown. We detected a high rate of membrane retrieval events in actin-rich lamellipodia and at the base of filopodia in the growth cone transitional domain, which is characterized by intense F-actin remodeling and membrane ruffling [41-43]. The formation of F-actin rich membrane ruffles correlates highly with endocytic activity in many cell systems, including phagocytosis in leukocytes [44], rac1-dependent pinocytosis in fibroblasts [45], membrane retrieval in the growth cone by endocytic vacuoles during basal outgrowth [19], and growth cone collapse induced by outgrowth inhibitory factors [16]. Conversely, F-actin depolymerization is necessary for Ca^2+^-induced macropinocytosis in the growth cone [46]. We, therefore, tested whether rapid membrane retrieval requires F-actin or, alternatively, is stimulated by F-actin disassembly. Treatment with cytochalasin D, which binds with high affinity to the barbed ends of F-actin and prevents filament elongation while allowing depolymeration from the pointed end [47,48], inhibited axon outgrowth in a dose-dependent manner (Figure 7A) as has been shown previously [49,50]. Treatment with lower dose cytochalasin D (30 nM) permitted a relatively normal rate of axon extension and growth cone motility yet significantly disrupted F-actin in the growth cone, as assessed by phalloidin staining (Figure 7A-B). We used this same condition to test how depolymerizing F-actin affected vesicle formation at sites of self-membrane contact in the growth cone, by utilizing the focal membrane labeling assay. Treatment with cytochalasin D (30 nM) reduced the formation of nascent endocytic vesicles at sites of cell-cell contact (Figure 7C). Significantly, both the percentage of self-membrane contact sites associated with the formation of nascent endocytic vesicles (Figure 7D, Additional file 10) and the frequency of membrane retrieval at self-membrane contact sites were reduced upon treatment with cytochalasin D compared to non-treated controls (Figure 7E). The overall number of self-membrane contacts observed during the focal membrane labeling assay tended to be lower after the cytochalasin D treatment but was statistically insignificant from the non-treated controls (Figure 7F). Taken together, these findings indicate that actin polymerization plays an important role in dynamic membrane retrieval by endocytic vesicles at sites of self-membrane contact in the growth cone.

Do endocytic hot-spots occur repeatedly at the same spatial locations or are they triggered within new areas of the same growth cone over time? To address this we developed a dual FM-dye labeling assay capable of monitoring the spatial profile of rapid membrane retrieval in the same growth cone at two different time-points (Figure 8A). The distinct emission wavelengths of FM 2-10 (620 nm) and FM 5-95 (734 nm) allowed for spectral separation during the sequential dye application in this assay. An initial pulse of FM 2-10 labeled nascent endocytic vesicles with similar spatial and temporal characteristics to that seen previously with FM 5-95, although FM 2-10 labeling was less efficient than FM 5-95 labeling (Figure 8B). A focal pulse of FM 5-95 from a second pipette, applied 40 s later, labeled a new set of nascent vesicles (Figure 8B'). Significantly, the endocytic hot-spots labeled by the second dye pulse (FM 5-95) were concentrated in distinct subdomains from the FM 2-10-positive vesicles that had formed just seconds prior (Figure 8B, B'' and Additional file 11). Thus, small vesicle endocytic hot-spots appear spatially and temporally stochastic in nature.

### Membrane retrieval by tubules and vacuoles

**Figure 9:**
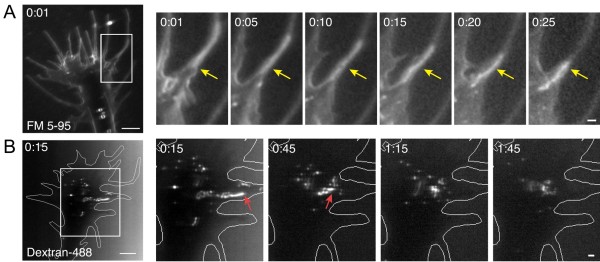
**Endocytosis of elongated tubules in the growth cone periphery**. **(A) **Time-lapse confocal images of FM 5-95 internalization show immediate dye incorporation into an elongated tubule (yellow static arrow) near the base of a filopodium. In subsequent frames, the endocytic tubule is transported retrogradely toward the growth cone central domain. The boxed area is magnified in the panels at the right and the time after the initial membrane labeling (minutes:seconds) is indicated in each frame. See Additional file 8. **(B) **Time-lapse confocal images of fluorescent dextran internalization show similar endocytosis of an elongated tubule (red arrows) near the base of a filopodium. The focal pulse (10 s) of fluorescent dextran was applied at 0:00 and the initial frame shows nascent endocytic vesicles immediately after the removal of uninternalized dextran (0:15). Endocytic vesicles originating in the peripheral domain move retrogradely and fuse with other dextran-containing vesicles near the transitional- or central domains of the growth cone within one minute. See Additional file 12. Scale bars, 5 μm (left), 1 μm (right).

In stark contrast to the small vesicles at endocytic hot-spots, we also observed elongated endocytic tubules in the growth cone periphery by the FM dye labeling assay. Endocytic tubules formed within or near the base of filopodia (Figure 9A, Additional file 8) and ranged from 1.1 to 7.8 μm in length (mean length: 4.4 ± 2.3 μm standard error of the mean, n = 8). Incorporation of FM dye into endocytic tubules corresponded temporally with surface membrane labeling, suggesting that these structures were contiguous with the plasma membrane at the time of focal dye application. We observed at least one dye-labeled endocytic tubule in 30% of the growth cones tested. Remarkably similar elongated tubules were also seen by the focal fluid-phase endocytosis assay (Figure 9B, Additional file 12), validating the endocytic nature of these compartments. Endocytic tubules in the growth cone periphery underwent processive retrograde movement toward the growth cone central domain at a rate of 10 μm/minute, consistent with reported rates for actin retrograde flow (see Discussion).

**Figure 10:**
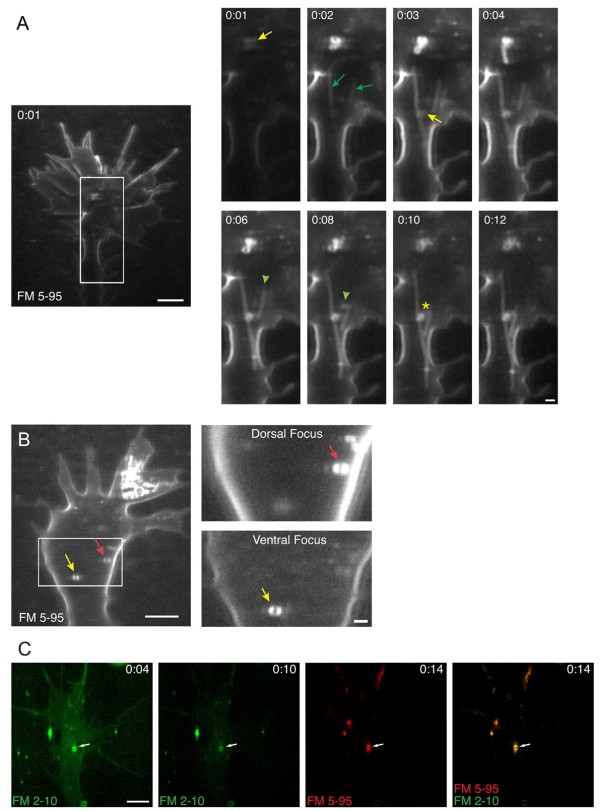
**Endocytic tubules and vacuoles in the growth cone central domain**. **(A) **Time-lapse confocal images of FM 5-95 internalization show dye incorporation into elongated tubules (green arrows, 0:02) and vacuoles (yellow arrows, 0:01 to 0:03) that correspond to the timing of surface membrane labeling. Tubules (green arrowheads, 0:06 to 0:08) and vacuoles are most often stationary but occasionally become motile and contact one another (yellow asterisk, 0:10 to 0:12). See Additional file 13 for the corresponding time-lapse movie. **(B) **Confocal images show FM 5-95 labeled vacuoles associated with dorsal (red arrow) and ventral (yellow arrow) surface membranes. The boxed region on the left is magnified at the right, which shows two different confocal z-sections of the same growth cone. Dye-labeled vacuoles are either seen in the dorsal (upper magnified panel) or ventral (lower magnified panel) confocal sections of the growth cone. The numerous vesicles in the upper-right lamellipodium of the far left panel originated from small vesicle hot-spots. Scale bars, 5 μm (left), 1 μm (right). **(C) **Time-lapse confocal images show that FM dye-labeled vacuoles are continuous with the plasma membrane for extended time periods. The time (minutes:seconds) following the initial dye pulse is indicated in each frame. In this modified dual-labeling assay, a first micropipette delivered a focal pulse of FM 2-10 at 0:00 (green, left panel), which rapidly labels several small vesicles and a vacuole (0:04, white arrow). A subsequent pulse of FM 5-95 (red), applied from a second micropipette at 0:12, incorporates into the same vacuole (0:14, white arrow). Scale bar, 5 μm.

Focal application of FM 5-95 also labeled elongated tubules in the growth cone central domain (Figure 10). Like the peripheral tubules, FM dye incorporated into the central tubules concomitantly with surface membrane labeling (Figure 10A, Additional file 13). The central tubules varied in length, in some instances extending as long as 12 μm through the growth cone body and into the axonal shaft. Whereas most central tubules were stationary, we occasionally observed rapid transport at rates exceeding 60 μm/minute (see Discussion). In some instances central tubules interacted with other FM dye containing compartments such as large vacuoles. Dye-labeled vacuoles were predominantly found in the central domain and were stationary with few exceptions. The labeled vacuoles ranged in size from 0.5 to 1.25 μm in diameter and had a visible lumen by confocal microscopy. Like the endocytic tubules, FM dye incorporation into vacuoles corresponded temporally with surface labeling, suggesting that these compartments were also contiguous with the plasma membrane at the time of focal dye application. By comparing multiple confocal z-sections, we found that vacuoles could be associated with either the ventral or dorsal surface membrane of the growth cone (Figure 10B). In total, central tubules and stationary vacuoles were observed in 50% and 85% of growth cones, respectively, during the brief focal endocytic assay.

Might FM dye-labeled vacuoles represent distinct endocytic compartments within the growth cone cytoplasm or are they contiguous with the plasma membrane? By performing the sequential FM dye membrane labeling assay, we found that in all instances (12/12), vacuoles immediately labeled by the initial FM 2-10 pulse also incorporated dye upon a subsequent FM 5-95 pulse (Figure 10C). In contrast, small endocytic vesicles labeled by the initial dye pulse were rarely labeled by the second dye pulse. These findings illuminate an important distinction between small endocytic vesicles and vacuolar structures. Small vesicles, which can incorporate both lipophilic and fluid-phase markers and exclude subsequent dye pulses, are rapidly internalized endocytic compartments. In contrast, vacuoles appear to be contiguous with the plasma membrane for extended durations that can be 10s of seconds to minutes. Although membrane-contiguous vacuoles may eventually undergo endocytosis, it is likely that the dye-labeled vacuoles seen by our membrane labeling assay rarely represent internalized compartments.

### Rapid recycling of internalized membrane

**Figure 11:**
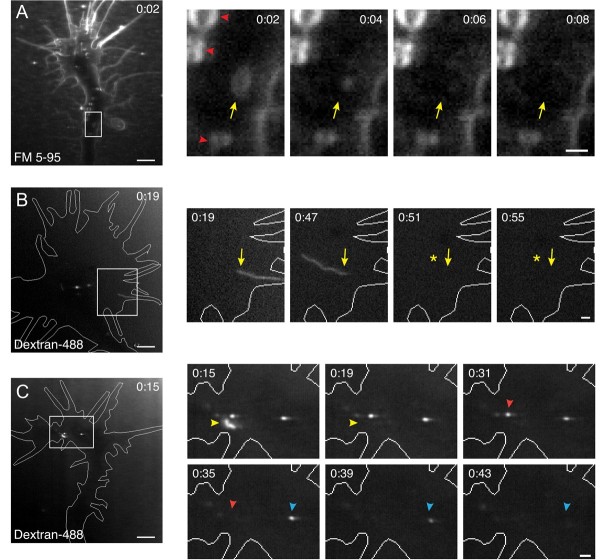
**Vacuole and tubule unloading or recycling back to the plasma membrane**. **(A) **Time-lapse confocal images of FM 5-95 internalization show the disappearance of a vacuole in the growth cone central domain. Focally applied FM 5-95 (applied at 0:00) immediately incorporates into several vacuoles (0:02). In subsequent frames, one vacuole narrows (yellow arrows, 0:04) and later disappears (0:06 to 0:08). Note that the size and location of other nearby vacuoles remains unchanged (red arrowheads). **(B) **Time-lapse confocal images of fluorescent dextran internalization show sudden dye unloading by an elongated tubule in the growth cone periphery (yellow static arrows). The dextran pulse was applied at 0:00 and washed away from 0:10 to 0:15. Note that the labeled tubule disappears within 40 to 50 s of forming (0:51, yellow asterisk). **(C) **Time-lapse confocal images show rapid unloading of dextran-containing vesicles. The focal pulse was applied and washed away as in (B). Within 20 to 30 s of vesicle formation, nascent vesicles (yellow, red, blue static arrowheads) begin to unload dye. For all examples (A-C), the boxed regions in the left panels are shown at higher magnification on the right and the time after the focal dye pulse (minutes:seconds) is indicated in each frame. Time-lapse movies for (A-C) are shown in Additional file 14. Scale bars, 5 μm (left), 1 μm (right).

What is the fate of internalized membrane? Most dye-labeled vacuoles were stationary and persisted in the growth cone for the duration of the focal endocytic assays. However, we observed the occasional constriction and rapid disappearance of dye labeled vacuoles in < 6% of all cases (Figure 11A, Additional file 14). To further monitor the subsequent transport and fate of nascent endocytic compartments, we performed the focal fluid-phase endocytic assay while extending the duration of time-lapse confocal imaging. As represented in Figure 9B, most of the labeled peripheral tubules and vesicles underwent centripetal retrograde transport toward the central domain of the growth cone. Within one to two minutes, tubules and vesicles fused with other dye-labeled compartments and constricted into small circular endosomes (Additional file 12). However, we also observed rapid dye unloading by peripheral tubules and smaller vesicles that occurred as early as 20 s after the initial endocytic internalization (Figure 11B, C, Additional file 14). The disappearance of endocytic vesicles, vacuoles and tubules may reflect rapid exocytic recycling of these compartments. Alternatively, the dye-labeled organelles may have fused with other non-labeled internal compartments, which could dilute the dye content below the level of fluorescence detection.

### Rates of membrane retrieval in central and peripheral regions of the growth cone

**Figure 12:**
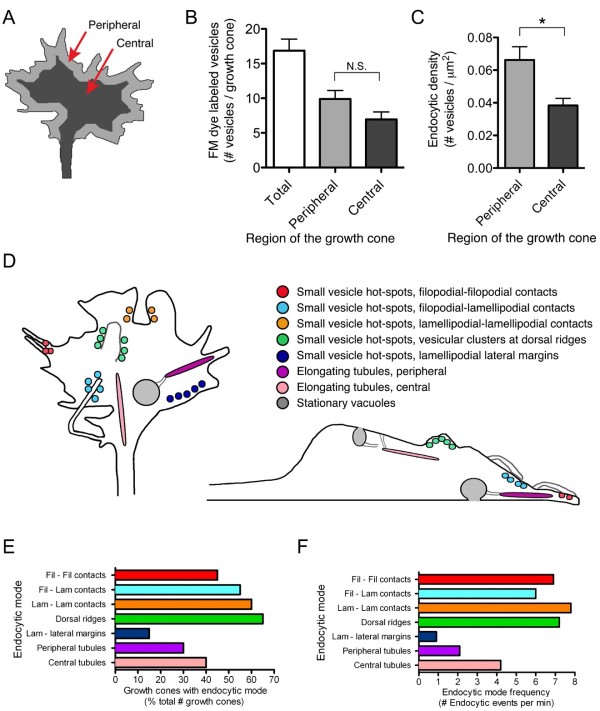
**Distribution of endocytosis in the growth cone central and peripheral domains**. **(A) **Schematic illustration of the growth cone peripheral and central regions (light and dark gray, respectively) used to determine the spatial distribution of endocytic vesicle formation **(B-C)**. (B) Summary of the number of endocytic events in the indicated regions. Individual vesicles were counted 15 s after membrane labeling and classified based on their origin in peripheral or central regions as defined in (A). Colors (light and dark gray) correspond to (A). (C) Summary of the endocytic density in peripheral and central regions as defined in (A). Vesicles were counted as in (B) and the density reflects the number of endocytic vesicles per μm^2^. Data are the mean ± standard error of the mean obtained from 20 individual growth cones. N.S., *P *> 0.05, *P < 0.005, *t*-test. (**D**) Summary illustration of the endocytic modes described in Figures 2-10. A top-view (coronal section) of the growth cone is shown at the left. A side-view (sagittal section) is shown at the right where dorsal is up and ventral is down. The colors of labeled vesicles, tubules, and vacuoles correspond to the legend at the upper right. **(E) **Summary of the percentage of growth cones that displayed individual endocytic modes during the focal membrane labeling assays. The colors for each bar correspond to the legend and illustration in (A). **(F) **Summary of the frequency of individual endocytic modes observed during the FM dye assays. Spatial modes were counted in 20 individual growth cones and are displayed as the number of events per minute.

We tested whether membrane retrieval is most prevalent in the peripheral or central regions of the growth cone by quantitating the number of FM dye-labeled structures that formed in each respective region (Figure 12A). This comparison revealed a similar number of total endocytic vesicles that form within central and peripheral regions (Figure 12B). However, we found that the density of vesicle formation differed between the central and peripheral domains (0.038 ± 0.004 standard error of the mean vesicles/μm^2 ^vs 0.066 ± 0.008 vesicles/μm^2^, respectively, Figure 12C). Taken together, these data suggest that the spatial properties of the described endocytic modes collectively specify the peripheral domain as the primary region of membrane retrieval. To gain further insight into membrane retrieval rates within central and peripheral domains, we next compared the frequency of individual endocytic modes (Figure 12D-F). Altogether, membrane retrieval at sites of self-membrane contact account for a large proportion of overall membrane retrieval (Figure 12E, F). These retrieval events, associated with growth cone peripheral processes, appear to be a driving force for the rapid membrane retrieval in the growth cone.

### Positive correlation between the rate of endocytosis and axon outgrowth

**Figure 13:**
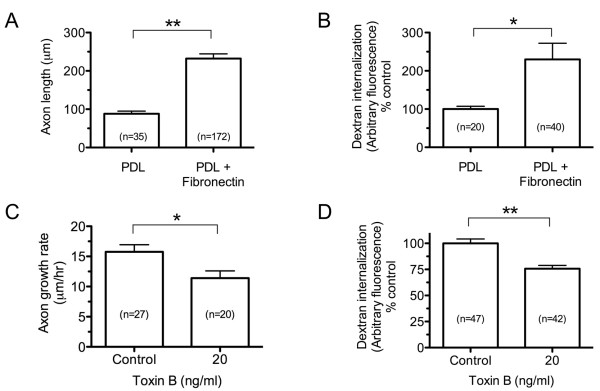
**Endocytic activity correlates with axon outgrowth and requires Rho GTPases**. **(A) **Summary of axon length measurements from spinal neuron cultures grown on poly-d-lysine (PDL) or PDL with fibronectin. **(B) **Summary of fluid-phase endocytosis levels measured in spinal neuron growth cones cultured on the indicated substrates. Fluorescent dextran was applied for 10 minutes and the amount of internalization was measured by fluorescence microscopy. The mean fluorescence intensity of each growth cone was normalized to the mean control (PDL). **(C) **Summary of growth rate measurements during the one-hour growth assays with or without pre-treatment with Toxin B (20 ng/ml). **(D) **Summary of fluid-phase endocytosis levels measured as in (B) under the indicated conditions. All data (A-D) are the mean ± standard error of the mean and the number of axons measured per condition is indicated in each bar. *P < 0.05, **P < 0.001, *t-*test or Mann-Whitney *U-*test (see Methods).

To gain insight into the functional role of endocytosis in growth cone motility, we next asked whether stimulating axon outgrowth with an extracellular matrix ligand could influence the rate of endocytosis in the growth cone. Plating embryonic *Xenopus *spinal neurons on a fibronectin substrate (PDL+FN) dramatically increased the rate of axon extension in comparison to poly-D-lysine (PDL) alone in an overnight outgrowth assay (Figure 13A). To determine whether substrate-stimulated growth cone motility affected the rate of membrane retrieval, we measured fluorescent dextran internalization in growth cones migrating on PDL or PDL+FN substrates. Positively correlating with the effects on axon outgrowth, fibronectin also stimulated the rate of fluid-phase endocytosis in the growth cone (Figure 13B).

To further probe the relationship between axonal growth rate and membrane retrieval, we next asked whether inhibiting Rho GTPases, which attenuates axon outgrowth, could affect the endocytic rate in the growth cone. Treatment of spinal neuron cultures with Toxin B, a general inhibitor of Rho GTPases [51], caused a modest but significant decrease in the rate of axon outgrowth (Figure 13C). Higher concentrations had a dramatic effect on substrate attachment and neurite formation that precluded their use (data not shown). Importantly, fluid-phase endocytosis was also inhibited significantly by Toxin B treatment (Figure 13D). Collectively, these findings provide further support for a positive correlation between the rate of axonal growth and membrane retrieval in the growth cone.

## Discussion

### Comparison of spatial endocytic modes: potential regulators and functions

The use of focal endocytic assays in this study has revealed the spatial dynamics of endocytic vesicle formation in actively extending growth cones for the first time. Using this approach, we have discovered several distinct modes of rapid membrane retrieval based on the spatial and temporal characteristics of vesicle formation, as summarized in Figure 12. Collectively, endocytic hot-spots elicited by self-contact between peripheral processes were the most frequent means of membrane retrieval. These include contacts among adjacent filopodia, lamellipodia, or between a filopodium and nearby lamellipodium. The disappearance of filopodia upon contact with the growth cone body or with other filopodia correlated with hot-spots of endocytic membrane retrieval. We found that vesicle formation at self-contact sites was sensitive to cytochalasin D treatment, implicating a role for F-actin polymerization in this process. Although the precise nature of these membrane remodeling events awaits further investigation, these observations suggest that membrane fusion and endocytic retrieval may represent additional mechanisms for removing filopodia and reshaping growth cone morphology. Furthermore, it is possible that these processes may allow spatially distant environmental information, at the tips of filopodia, to be rapidly transmitted to the growth cone body. For instance, receptor activation and second messenger signals in the filopodia may be relayed to the growth cone, cooperating with previously reported means for spatial transmission of second messenger signals [52,53].

Small-vesicle hot-spots at membrane ridges were the second most frequently observed endocytic route. This includes vesicle formation at dorsal ridges, as well as ridges that form at the lateral margins of lamellipodia as the growth cone changes shape. These regions undergo considerable membrane remodeling and are very transient in nature, forming and disappearing within seconds. It is likely that actin dynamics and membrane curvature are integrally involved in ridge formation, ruffling and vesicle formation. The actin regulator Rac1 and the WAVE complex have been closely linked with forms of macropinocytosis [16,45,54]. Furthermore, BAR-domain proteins, which facilitate membrane curvature, also participate in membrane ruffling and macropinocytosis [55]. Endocytic hot-spots within membrane ridges at the dorsal surface and lateral margins of the growth cone may be regulated by these same effectors.

We observed an abundance of large stationary vacuoles in the growth cone central domain. Most vacuoles incorporated FM dye immediately upon surface membrane labeling, suggesting that these structures were pre-existing and continuous with the plasma membrane at the time of labeling. This conclusion was further supported by our sequential FM dye labeling experiments, which showed that the same vacuolar structures could be labeled by dual dye pulses separated by 40 seconds. It is likely that the vacuoles seen in this study are synonymous to reverse shadow-cast vacuoles previously observed by correlative DIC and electron microscopy, which often contained an orifice that contacted the plasma membrane [35]. By tracking vacuoles with DIC video microscopy, Dailey and Bridgman also found that vacuoles in the growth cone central domain were much more stable (10- to 20-minute lifetime) than endocytic compartments that formed in the peripheral domain.

Finally, we also found evidence for internalization and rapid recycling of elongated tubules in both the central and peripheral regions of the growth cone. Although tubule formation was relatively infrequent, the size of these compartments (up to 12 μm in length) suggests that tubules account for the majority of bulk membrane retrieval by surface area. This process may be similar to the high-capacity membrane retrieval system described in non-neuronal cells, which is regulated by Cdc42 and GRAF1 and may facilitate adhesion receptor recycling [56-58]. The focal endocytic assays used in the present study provided direct observation of endocytic tubules within seconds after internalization. This rapid detection was critical, because peripheral tubules typically fused with other compartments or were recycled within one to two minutes. In all instances, nascent peripheral tubules were processively transported toward the central domain of the growth cone at rates near 10 μm/minute. This is comparable to the reported rates for actin retrograde flow, measured at 5 to 6 μm/minute in cultured neurons isolated from other species [59-61]. In contrast, central tubules and vacuoles were most often stationary, but the infrequent transport of these structures proceeded at more rapid rates consistent with microtubule-based axonal transport [62,63].

### Use of FM dyes to monitor endocytosis in the growth cone

The use of FM dyes to monitor vesicle dynamics in neurons was pioneered by Betz and colleagues in the 1990s and has since been utilized by numerous studies that have greatly advanced our understanding of synaptic function [64]. Membrane labeling with FM dyes has also been used in the growth cone in order to track the fate of endocytic compartments [11,18,19,37]. In this study, we have optimized an approach to rapidly image the initial formation and early trafficking of nascent endocytic structures locally within the growth cone. The ability of this technique to reveal single vesicle formation with high spatial and temporal resolution is likely due to three main attributes of this assay. First, the fluorescence emission of FM dyes is at least two orders of magnitude brighter when bound to the plasma membrane than in aqueous solution. Second, the brief focal dye pulse allows the free FM dye that remains non-bound to the surface membrane to be rapidly diluted into the surrounding buffered saline. Furthermore, FM 5-95 and FM 2-10 are slightly less lipophilic than the more commonly used FM 1-43 and FM 4-64, and consequently de-stain from the plasma membrane in a relatively quick manner [65], allowing the transient labeling and imaging of rapid membrane retrieval events. In our hands, FM 2-10 de-stained even more rapidly than FM 5-95.

The amphiphilic nature of FM dyes implies that they bind to the plasma membrane, are internalized by vesicular processes, and become trapped in nascent cytoplasmic vesicles as the dye is unable to cross the lipid bilayer. Recent studies have validated the ability of FM dyes to selectively label endocytic vesicles. First, fluorescence resonance energy transfer (FRET) studies detected no interaction between membrane-bound FM dyes and a cytoplasmic-GFP under physiological conditions [66]. Furthermore, FM dyes microinjected into the cytoplasm of cells fail to label intracellular organelles and vesicles [67]. Thus, the dye labeling emanating from intracellular membranes is unlikely to come from dye that was somehow able to traverse the plasma membrane.

Our use of the focal membrane labeling assay, combined with confocal microscopy, demonstrates that structures associated with both the dorsal and ventral (apical and basal) surface membranes can be labeled (Figure 10B). However, due to the close apposition of the ventral membrane with the underlying substrate, it is possible that dye labeling is non-uniform. Our own findings, combining the focal labeling assay with total internal reflection microscopy (TIRF), support this notion, as ventral membrane labeling lags slightly behind the dorsal surface (data not shown). Therefore, this assay may be inherently biased toward measuring membrane retrieval at the apical surface of the growth cone. This property, which could be a potential advantage or impediment depending on the assay, should be considered by investigators utilizing the focal membrane labeling assay in future studies.

### Functions for high-capacity membrane retrieval and recycling systems

Taken together, the present findings provide further insight into rapid and high capacity membrane retrieval and recycling systems in the growth cone. Although the functions of these energetically demanding processes are yet to be understood, similar membrane recycling systems in non-neuronal cells appear to be driving factors for cell polarization. For example, in migrating fibroblasts, clathrin-independent carriers (CLICs) internalize the vast majority of membrane and extracellular fluid at the leading edge. This recently defined endocytic mechanism, previously considered macropinocytotic, is now recognized to enrich specific molecular cargo such as the adhesion proteins β1-integrin, Thy-1 and CD44, and is critical for optimal cell migration [56,57]. Furthermore, clathrin-mediated endocytosis of specific cargo is also polarized to the front of migrating cells. For instance, the endocytic adaptor proteins Numb, Dab2, and ARH cooperatively facilitate endocytosis of integrin receptors, which need to be subsequently recycled in order to polarize focal adhesion turnover to the leading edge [68-71]. Similar vesicular processes can spatially localize cytoskeletal activity (Rac1, Cdc42) and receptor- and nonreceptor- tyrosine kinase signaling to the leading edge of migrating cells [27,29,72,73].

In the growth cone, cytoskeletal protrusion, adhesion complex turnover, and tyrosine kinase signaling are all polarized to the leading edge [74,75]. It is possible that one or more of the endocytic modes described in this study contribute to these processes in order to optimize axon extension. In an over-simplified model, membrane addition (exocytosis) would facilitate axon extension, whereas membrane retrieval (endocytosis) would attenuate extension by removing bulk membrane. However, this model is contradicted by recent findings that show the rate of endocytic membrane retrieval positively correlates with the dynamic remodeling of growth cone shape [19]. The results of the present study further extend this notion by showing that substrate-stimulated outgrowth also correlates positively with increased endocytic membrane retrieval. Local modes of endocytosis may promote axon outgrowth by polarizing signaling, cytoskeletal dynamics or adhesion turnover to the leading edge. Further characterization of endocytic routes in the growth cone will enable the rigorous testing of these models in future studies.

## Conclusions

In this study, we have utilized live-cell confocal microscopy and a transient membrane-labeling assay to reveal the spatiotemporal dynamics of rapid membrane retrieval and turnover in extending spinal neuron growth cones. This approach demonstrated that endocytic events are stochastic and occur at hot-spots initiated at sites of active membrane remodeling or self-contact between peripheral extensions of the growth cone, with unique spatial and temporal properties. The rate of these bulk endocytic processes correlates with the rate of axon outgrowth and requires the function of Rho-family GTPases, suggesting that one or more distinct endocytic modes has important roles in growth cone motility. Future characterization of the molecular regulators and functional cargo associated with these endocytic modes will uncover the functional contributions of these processes to growth cone motility and chemotactic guidance.

## Methods

### Primary neuron culture and immunofluorescence labeling

Spinal neuron cultures from stage 22 *Xenopus laevis *(Xenopus 1, Dexter, MI, USA) embryos of either sex were prepared by methods previously described [76,77]. All experiments and animal housing were conducted according to National Institutes of Health (NIH, Bethesda, MD, USA) guidelines for animal care and safety, with the approval and under the auspices of the Mayo Clinic Institutional Animal Care and Use Committee. Unless indicated, spinal neuron cultures were grown on non-coated coverglass at room temperature (20 to 22°C) and experiments were performed 12 to 20 h after plating. Coating with poly-D-lysine (PDL, 0.5 mg/ml; Sigma, St. Louis, MO, USA) and fibronectin (FN, 20 μg/ml; Sigma) was performed in Dulbecco's phosphate-buffered saline (D-PBS) for one hour (37°C) followed by repeated washes in calcium- and magnesium-free PBS. Culture medium consisted of Leibovitz medium (87.1% vol/vol, GIBCO, Grand Island, NY, USA), fetal bovine serum (0.4% vol/vol, HyClone, Logan, UT, USA), and saline solution (12.5% vol/vol; 10 mM D-glucose, 5 mM sodium pyruvate, 1.26 mM calcium chloride (CaCl_2_), and 32 mM HEPES, pH 7.5). Cultured spinal neurons were chemically fixed (20 minutes; 2.5% formaldehyde, 0.01% glutaraldehyde), permeabilized with Triton X-100 (0.1%) and processed for immunofluorescence labeling as described [12,78]. Microtubules were labeled with polyclonal anti-β-tubulin (0.4 μg/ml; Abcam, Cambridge, England, UK) and an Alexa488 conjugated secondary antibody (2 μg/ml; Invitrogen, Carlsbad, CA, USA). Filamentous actin was labeled with Alexa555-conjugated phalloidin (260 nM; Invitrogen).

### Image acquisition and processing

We acquired digital time-lapse DIC images using a Zeiss (Jena, Germany) Axiocam CCD camera mounted on a Zeiss Axiovert 200 M inverted microscope (100 × oil immersion objective, 1.4 numerical aperture, 1.6 × optical zoom). For rapid time-lapse imaging of endocytic membrane retrieval, we used a Zeiss LSM 5LIVE confocal microscope equipped with a 63 × water immersion objective (1.2 numerical aperture, 2 × optical zoom). Individual frames were acquired at a rate of 100 ms per capture. We generated all representative movies using Image J software (NIH, LSM toolbox plugin) by exporting time-lapse stacks to a QuickTime format (MOV, MPEG4 compression, three frames per second) [78]. Images of immunolabeled growth cones were captured on a Zeiss LSM 5LIVE confocal microscope using a 63 × oil immersion objective (1.4 numerical aperture, 1.6 × optical zoom).

### Focal endocytic assays

All focal endocytic assays were performed in a serum-free modified Ringers (MR) solution (120 mM sodium chloride (NaCl), 2.2 mM potassium chloride (KCl), 2 mM CaCl_2_, 1 mM magnesium chloride (MgCl_2_), 5 mM HEPES, 2 mM sodium pyruvate; pH 7.6, 20 to 22°C). Spinal neuron cultures on glass-bottomed uncoated dishes were positioned over an inverted confocal microscope. Using a micromanipulator stabilized by a floatation table, we positioned a micropipette 100 μm in front of the leading edge of the growth cone in the direction of neurite extension. We fabricated micropipettes to an approximate 1-μm opening by heat-pulling capillary glass (1 mm OD, 0.58 mm ID, Warner Instruments, Hamden, CT, USA) with a micropipette puller (Flaming/Brown, Sutter Instruments, Novato, CA, USA; and PC-10, Narishige, East Meadow, NY, USA). A stock solution of FM 5-95 or FM 2-10 (10 mM in H_2_0, Invitrogen) was diluted to 1 mM or 2 mM, respectively, in MR and 2 to 4 μL were loaded into each micropipette. A picospritzer (Picospritzer III, Parker Instrumentation, Huntsville, AL, USA) controlled focal dye application by applying four repetitive pulses (2 Hz, 400 ms pulse duration, 2.5 p.s.i.) immediately after the onset of confocal imaging. Cytochalasin D (30 nM; Sigma) was added 30 minutes before dye application and confocal imaging. For focal application of fluorescent dextran, the micropipette was loaded with fluorophore-conjugated dextran (Alexa488 or tetramethylrhodamine-labeled, 10,000 MW, neutral charge, Invitrogen; 1 mM in MR) and positioned 80 μm in front of the growth cone in the direction of neurite extension. A second micropipette containing MR was used to focally wash away uninternalized dextran. A picospritzer controlled both micropipettes by delivering 10 to 20 repetitive pulses of fluorescent dextran (2 Hz, 120 ms duration, 2.5 p.s.i.) and subsequently washing away uninternalized dextran with the second micropipette (2 Hz, 120 ms duration, 2.5 p.s.i.) until the background fluorescence intensity subsided and internal vesicles could be visualized (approximately 5 to 10 s). For co-internalization of FM 5-95 (100 μM in the micropipette) and fluorescent dextran (Alexa-488 conjugated; 500 μM in the micropipette), we simultaneously applied both dyes for 10 s from the same micropipette (2 Hz, 120 ms duration, 2.5 p.s.i.). A second micropipette was used to wash away uninternalized fluorescent dextran as previously described.

### Determination of endocytic density

We determined the distribution of endocytic vesicles in the peripheral and central regions of the growth cone by counting individual vesicles within the defined regions of interest. All analyses were performed within ImageJ software (Bio-Formats ZVI plug-in, Madison, WI, USA). Individual vesicles were identified 15 s after the initial focal FM 5-95 application. Vesicles that had originated within 1 μm of the outline of the growth cone were considered peripheral. In order to determine the area of individual growth cones, we set fluorescence thresholds slightly above the background fluorescence levels and generated binary images (background fluorescence = 0, membrane fluorescence = 1). We then selected the outline of the growth cone as a region of interest in order to measure the total area. In order to determine the area of the central domain, we eroded the peripheral region of the binary growth cone image (1-μm diameter), redefined the region of interest outlining the new growth cone (central region), and measured the area within. The area of the peripheral region was determined by subtracting the central area from the total growth cone area. Endocytic density values were determined by dividing the number of endocytic events by the area of the respective region. Data from multiple growth cones was then averaged to determine the mean endocytic density (the number of vesicles per μm^2^).

### Quantitative fluid-phase endocytic assay

For comparisons of the rate of membrane retrieval, we incubated spinal neuron cultures with fluorescent dextran (150 μM; Texas Red conjugated, 3000 MW, lysine fixable, Invitrogen) for 10 minutes at room temperature followed by consecutive rinses (10 minutes) at reduced temperature (10°C). Neurons were then chemically fixed with 5% formaldehyde in a cytoskeleton-stabilizing buffer for 20 minutes and mounted with Prolong Gold (Invitrogen) [78]. *Clostridium difficile *Toxin B (20 ng/ml, Calbiochem, Gibbstown, NJ, USA) was applied at the time of plating. Culture medium was used for all dye incubations and washes. We acquired digital fluorescence images using a Zeiss Axiocam mounted on a Zeiss Axiovert 200 M inverted microscope (20 ×, 0.8 numerical aperture, 1.6 × optical zoom). Identical acquisition parameters were used for all experimental groups and the original 14-bit images were analyzed using ImageJ software. A region of interest encompassing the entire growth cone (defined as the distal 40 μm of the axon) was used to determine the mean fluorescence intensity of dextran-labeled endocytic vesicles in the growth cone. A threshold was set above the background intensity, identical for all conditions, and the fluorescence intensity of the region of interest was measured. Data were background subtracted and the final corrected intensity value for each growth cone was normalized to the appropriate mean control.

### Axonal growth assays

To determine the effect of increasing doses of cytochalasin D on axon outgrowth, we measured the rate of axon extension during a 1-h assay performed 12 to 14 h after plating. Cytochalasin D (10 to 100 nM) or dimethyl sulfoxide (DMSO) was added 30 minutes prior to the growth assay. For measurements of neurite length on different substrates, spinal neurons were plated on PDL or PDL + FN substrates. After 14 h *in vitro*, cultures were chemically fixed and phase-contrast digital images were captured using a cooled CCD camera (ProgRes C10 plus, Jenoptik, Jupiter, FL, USA) mounted on a Zeiss (Axiovert 40CFL) inverted microscope (10 × objective). Axon lengths were determined using the ImageJ plug-in NeuronJ [79]. We measured only the longest neurite, or branch of each neurite, and only axons > 50 μm in length were included in the analysis. To determine the effect of Toxin B on axon outgrowth, we measured the rate of axon extension during a 1-h assay performed 12 to 20 h after plating [78]. Toxin B (20 ng/ml) was added at the time of plating.

### Statistical analyses

Statistical analyses were performed using Graphpad Prism software (v5, La Jolla, CA, USA). The D'Agostino and Pearson omnibus test was used to assess the data for normality. Statistical comparisons with normal distributions used either a two-tailed *t-*test or one-way analysis of variance (ANOVA; Tukey post-test), as indicated in the figure legends. All other comparisons utilized the non-parametric Mann-Whitney *U*-test.

## Abbreviations

CLICs: clathrin-independent carriers; CytoD: cytochalasin D; DIC imaging: differential interference contrast imaging; D-PBS: Dulbecco's phosphate-buffered saline; DMSO: dimethyl sulfoxide; ECM: extracellular matrix; F-actin: filamentous actin; FN: fibronectin; FRET: fluorescence resonance energy transfer; MR: modified Ringers; PDL: poly-D-lysine; PI3K: phosphoinositide 3-kinase; TIRF microscopy: total internal reflection fluorescence microscopy; Toxin B: *Clostridium difficile *toxin B

## Competing interests

The authors declare that they have no competing interests.

## Authors' contributions

JHH and JRH conceived the project and designed experiments. JHH, SJH, LPC and MA-R performed experiments and analyzed data. JHH and JRH wrote the manuscript. JRH supervised the project. All authors read and approved the final manuscript.

## Supplementary Material

Additional file 1 **Movie 1 - Small endocytic vesicle hot-spots at dorsal ridges of the growth cone**. These representative time-lapse movies show examples of rapid membrane retrieval triggered at dorsal ridges. A focal pulse of FM 5-95 transiently labeled the plasma membrane (evident at 00:00). The brief dye pulse was delivered from a micropipette positioned 100 μm in front of the leading edge of the growth cone, which is indicated by the top arrow in frame 1. Confocal images were collected (1 Hz) during the endocytic assay and the time frames (minutes:seconds) are indicated at the top left. Movie 1 corresponds to Figure 2A, B. The scale bar (5 μm) applies to each example. Format: MOV (MPEG4 compression). Click here for file

Additional file 2 **Movie 2 - Small endocytic vesicle hot-spots at the lateral margins of lamellipodia**. This representative time-lapse movie demonstrates membrane retrieval triggered at the lateral margins of a lamellipodium. A focal FM 5-95 pulse transiently labelled the growth cone surface membrane and confocal images were acquired as described in Movie 1. The yellow static arrow (00:16) indicates a region undergoing active membrane remodelling and retrieval at the lateral-most lamellipodial margin. Movie 2 corresponds to Figure 3A. Scale bar, 5 μm. Format: MOV (MPEG4 compression). Click here for file

Additional file 3 **Movie 3 - Small endocytic vesicle hot-spots at the lateral margins of lamellipodia**. This representative time-lapse movie shows membrane remodelling at the lateral margins of the growth cone lamellipodium (black static arrow). The DIC images were acquired at 0.5 Hz and the time (minutes:seconds) is indicated at the top left. Movie 3 corresponds to Figure 3B. Scale bar, 5 μm. Format: MOV (MPEG4 compression). Click here for file

Additional file 4 **Movie 4 - Small endocytic vesicle hot-spots triggered by filopodial - lamellipodial contacts**. This representative time-lapse movie demonstrates membrane retrieval triggered by contact between a filopodium and lamellipodium. A focal pulse of FM 5-95 transiently labeled the growth cone surface membrane and confocal images were acquired as described in Movie 1. The yellow arrow (00:09) indicates filopodial detachment, subsequent contact with the growth cone dorsal surface, and the formation of numerous small endocytic vesicles (00:21 to 00:27). Movie 4 corresponds to Figure 4A. Scale bar, 5 μm. Format: MOV (MPEG4 compression). Click here for file

Additional file 5 **Movie 5 - Small endocytic vesicle hot-spots triggered by filopodial - lamellipodial contacts**. This time-lapse movie shows evidence for the disappearance of growth cone filopodia following contact with an adjacent lamellipodium (black arrows, 00:00 to 00:26, 00:40 to 01:08). The DIC images were acquired at 0.5 Hz and the time (minutes:seconds) is indicated at the top left. Movie 5 corresponds to Figure 4B. Scale bar, 5 μm. Format: MOV (MPEG4 compression). Click here for file

Additional file 6 **Movie 6 - Small endocytic vesicle hot-spots triggered by filopodial contact**. This representative time-lapse movie shows membrane retrieval triggered by contact between neighboring filopodia (yellow arrowheads). A focal pulse of FM 5-95 transiently labeled the growth cone surface membrane and confocal images were acquired as described in Movie 1. The blue arrow points toward nascent endocytic vesicles formed at the base of filopodia. Movie 6 corresponds to Figure 5A. Scale bar, 5 μm. Format: MOV (MPEG4 compression). Click here for file

Additional file 7 **Movie 7 - Small endocytic vesicle hot-spots triggered by filopodial contact**. This time-lapse movie demonstrates contact between adjacent filopodia (black static arrow) similar to that shown by fluorescence images in Additional file 6. The DIC images were acquired at 0.5 Hz and the time (minutes:seconds) is indicated at the top left. Movie 7 corresponds to Figure 5B. Scale bar, 5 μm. Format: MOV (MPEG4 compression). Click here for file

Additional file 8 **Movie 8 - Membrane retrieval via lamellipodial contact and peripheral tubules**. This representative time-lapse movie demonstrates membrane retrieval triggered upon contact between adjacent regions of lamelipodium (Figure 6A), indicated by the yellow static arrow (00:00 to 00:12). In later frames, an elongated tubule (Figure 9A) is retrieved from a filopodium (blue static arrowhead, 00:07 to- 00:27). Surface labeling and image acquisition was performed as described in Movie 1. Scale bar, 5 μm. Movie 8 corresponds to Figure 6A and 9A. Scale bar, 5 μm. Format: MOV (MPEG4 compression). Click here for file

Additional file 9 **Movie 9 - Examples of membrane remodeling at sites of lamellipodial - lamellipodial contact**. This time-lapse DIC movie shows examples of membrane rearrangements at lamellipodial contacts, similar in nature to those shown in Movie 8. Movie 9 corresponds to Figure 6C and 6 the time (minutes:seconds) is indicated at the top left. The time 00:00 corresponds to 0:00 in Figure 6C. The time 03:34 corresponds to 0:00 in Figure 6B. During each respective time period, the black arrow points toward the region of interest highlighted in Figure 6B, whereas the white arrowheads point toward reverse shadowcast structures (00:17 to 00:25) identified in Figure 6C. Scale bar, 5 μm. Format: MOV (MPEG4 compression). Click here for file

Additional file 10 **Movie 10 - Endocytic vesicle formation at self-membrane contacts requires F-actin**. This time-lapse movie shows a representative example of the focal membrane labeling assay performed on a growth cone pre-treated with cytochalasin D (30 nM). Movie 10 corresponds to Figure 7C and 7 the time (minutes:seconds) is indicated at the top left. A filopodium contacts a nearby region of the growth cone periphery (yellow arrow, 00:21 to 00:26). This self-contact fails to elicit vesicle formation at this location. Surface labeling and image acquisition was performed as described in Movie 1. Scale bar, 5 μm. Format: MOV (MPEG4 compression). Click here for file

Additional file 11 **Movie 11 - Sequential dye labeling demonstrates temporally distinct endocytic zones**. This time-lapse movie shows that endocytic hot-spots at two separate time-points occur at spatially distinct areas of the same growth cone. The dual FM dye labelling assay was performed as described in Figure 8A. Briefly, a focal pulse of FM 2-10 (green) was applied at 00:00. A second micropipette then applied a subsequent focal pulse of FM 5-95 at 00:38. Movie 11 corresponds to Figure 8B-B''. The time (minutes:seconds) is indicated at the top left. Scale bar, 5 μm. Format: MOV (MPEG4 compression). Click here for file

Additional file 12 **Movie 12 - Membrane retrieval by elongated tubules in the growth cone periphery**. This time-lapse movie demonstrates membrane retrieval by elongated tubules in the growth cone periphery. Alexa-488 dextran was focally applied from a micropipette positioned 80 μm in front of the leading edge of the growth cone (top arrow in frame 1). Uninternalized dextran was then rapidly washed away during image acquisition using a second micropipette containing buffered saline (left arrow in frame 1). The time (minutes:seconds) is indicated at the top left. Movie 12 corresponds to Figure 9B. Scale bar, 5 μm. Format: MOV (MPEG4 compression). Click here for file

Additional file 13 **Movie 13 - Endocytic tubules and vacuoles in the growth cone central domain**. This time-lapse movie demonstrates rapid labeling of elongated tubules and vacuoles within the growth cone central domain. A focal pulse of FM 5-95 transiently labeled the growth cone surface membrane and confocal images were acquired as described in Movie 1. The yellow static arrowhead indicates a central vacuole and the green arrows point toward central tubules. Scale bar, 5 μm. Movie 13 corresponds to Figure 10A. Format: MOV (MPEG4 compression). Click here for file

Additional file 14 **Movie 14 - Vacuole and tubule unloading or recycling back to the plasma membrane**. These representative time-lapse movies correspond to Figure 11A-C. The boxed regions at left are shown at higher magnification on the right. The upper time-lapse series was performed using the focal FM 5-95 labeling assay, whereas fluorescent dextran was focally applied to growth cones in the center and lower series (indicated in frame 1). Scale bars, 5 μm (left), 1 μm (right). Format: MOV (MPEG4 compression). Click here for file
